# Adiponectin regulates BMSC osteogenic differentiation and osteogenesis through the Wnt/β-catenin pathway

**DOI:** 10.1038/s41598-017-03899-z

**Published:** 2017-06-16

**Authors:** Yiyao Wang, Xiaohui Zhang, Jun Shao, Hanghang Liu, Xian Liu, En Luo

**Affiliations:** 1 State Key Laboratory of Oral Diseases, National Clinical Research Center for Oral Diseases, West China Hospital of Stomatology, Sichuan University, Chengdu, 610041 People’s Republic of China; 2 Department of Stomatology, Guangzhou Hospital of Integrated Traditional and Western Medicine, Guangzhou, 510800 People’s Republic of China

## Abstract

Recent studies have demonstrated the stimulatory effects of adiponectin on bone formation, but the mechanism underlying these effects remains unclear. The Wnt/β-catenin pathway, one of the most important pathways in osteogenesis, has rarely been associated with the osteogenic effects of adiponectin in previous studies. The present study was designed to investigate the effects of adiponectin on bone mesenchymal stem cell (BMSC) osteogenic differentiation and bone formation through the Wnt/β-catenin pathway. We detected adiponectin receptor expression in BMSCs, constructed a recombinant adenovirus containing the human adiponectin gene, and then used the adenovirus to transfect BMSCs *in vitro* or injected the adenovirus into bone defect areas in animal models. Wnt/β-catenin pathway and osteogenesis were detected by real-time PCR, western blotting, immunofluorescence, HE staining and micro-CT. In both our *in vivo* and *in vitro* experiments, we detected higher gene and protein expression levels of the Wnt/β-catenin pathway-related factors β-catenin and cyclinD1 in adiponectin transgenic BMSCs and rats. Similar results were noted regarding the gene and protein expression levels of osteogenesis-related genes. In addition, more new bone formation was observed in the adiponectin-treated groups. Our results indicate that adiponectin could facilitate BMSC osteogenic differentiation and osteogenesis, and the Wnt/β-catenin pathway was involved in the osteogenic effect of adiponectin.

## Introduction

Adipose tissue, which has been regarded as a simple lipid storage organ in the past, has since become known as a vital endocrine system^1^. It secretes many cytokines that take part in a series of pathophysiological process^2^. Leptin is the first fat cytokine to be found to join the metabolism of bone. It can inhibit bone formation through the central regulation of the hypothalamus, or increase the osteoprotegerin and inhibit the differentiation of osteoclast through peripheral pathway^3, 4^. Visfatin, secreted by visceral fat cells, can also improve osteoblast differentiation and mineralization, and inhibit the production of osteoclasts^5^. Growing evidences suggest that adipose tissue is closely related to the regulation of bone metabolism.

Adiponectin (ApN), one of the adipocytokines, plays a critical role in glucose and lipid metabolism, anti-inflammation and insulin resistance attenuation^6–8^. Two ApN receptors were initially identified by Yamauchi. AdipoR1 is predominantly expressed in muscle, and AdipoR2 is predominantly expressed in the liver^9^. These differences in the distributions of the two adiponectin receptors increase the precision and effectiveness of ApN. Berner *et al*. found that ApN and AdipoR1 are expressed in human osteoblasts and mouse MC3T3-E1 cells^10^. Shinoda and Lee subsequently detected the expression of ApN and its receptors in cells of osteoblastic and osteoclastic lineages^11, 12^. Growing evidence suggests that a close relationship exists between ApN and bone metabolism, implying that ApN may play a direct role in bone metabolism by binding to its receptors^13^.

The role of ApN in the promotion or inhibition of bone metabolism remains controversial. Lower bone mineral density (BMD) has been noted in elderly men with high circulating ApN levels^14^. Fewer osteoclasts and higher BMD were observed in ApN-deficient mice than in wild-type mice^15^. In contrast, Jiang *et al*. discovered that intermittent ApN administration can promote bone formation and mineralization in rabbits with mandibular osteodistraction^16^, and Wu *et al*. showed that ApN can suppress sympathetic tone and increase bone mass in mice^17^. Luo *et al*. found that sustained ApN release can promote peri-implant osteogenesis in ovariectomization rabbits by suppressing osteoclasts^18^. ApN can increase bone mass by suppressing osteoclastogenesis and bone resorption^19, 20^, stimulating local angiogenesis^21, 22^, or promoting osteogenic differentiation^19, 23–27^. Several osteogenesis signalling pathways have been found to be activated in the presence of ApN; however, few studies have discussed the relevance of ApN and the Wnt/β-catenin pathway with respect to osteogenesis.

The Wnt/β-catenin pathway, also known as the canonical Wnt pathway, was found to be strongly linked with bone formation^28, 29^. Wnt protein acts on its cell-surface receptor to prevent β-catenin degradation^30^. Then, the up-regulated β-catenin translocates into the nucleus and stimulates the expression of various downstream genes^31^. The Wnt/β-catenin pathway has been demonstrated to promote BMSC osteogenic differentiation^32–34^. It is noteworthy that reduced bone formation and decreased β-catenin expression levels were observed in AdipoR1-knockdown BMSCs compared with wild-type BMSCs^25^. Accordingly, we deduced that ApN can act on BMSCs through the Wnt/β-catenin pathway.

Given the above evidence, we aimed to study the osteogenic effects of ApN through the Wnt/β-catenin pathway in ApN gene-modified BMSCs and rat tibia bone defect model. The following hypotheses were tested: (1) ApN receptors are expressed in BMSCs, (2) ApN can activate the Wnt/β-catenin pathway and promote gene-modified BMSC osteogenic differentiation and bone formation, and (3) ApN can activate the Wnt/β-catenin pathway and promote osteogenesis in transgenic mice.

## Results

### Characterization of BMSCs

**Figure 1:**
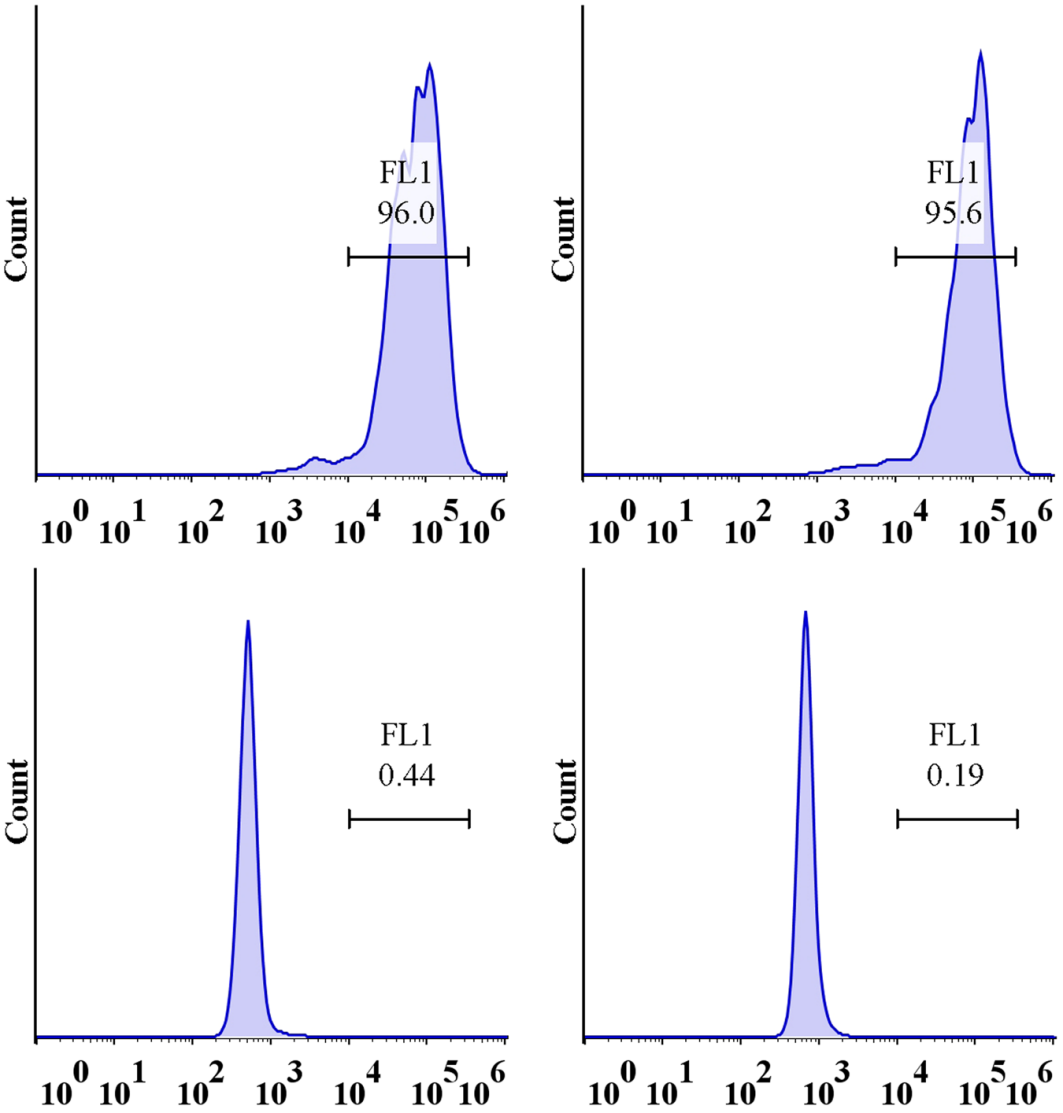
The cell surface antigens of BMSCs were detected by flowcytometer. Positive expression of CD29+ (96.2%), CD90+ (95.9%) and negative expression of CD34− (0.38%), CD45− (0.18%) were found.

The third-passage BMSCs were a homogenous population and exhibited a spindle-shaped fibroblastic morphology. The flow cytometry results showed positive CD29+ (96.0%) and CD90+ (95.6%) expression and negative CD34− (0.44%) and CD45− (0.19%) expression (Fig. 1), indicating that the BMSCs were of high purity. Passage 3 BMSCs were used for transfection.

### Identification of adenovirus transfection

**Figure 2:**
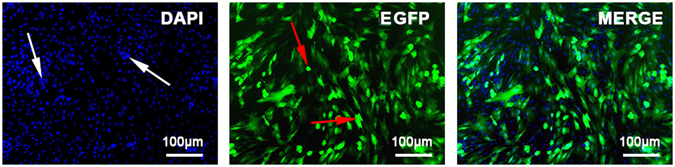
Immunofluorescence images of Ad-hAPN-EGFP (red arrow) in BMSCs. After transfection for 36 h, cell nuclei were stained with DAPI (white arrow). The transfection efficiency was up to 95%.

**Figure 3:**
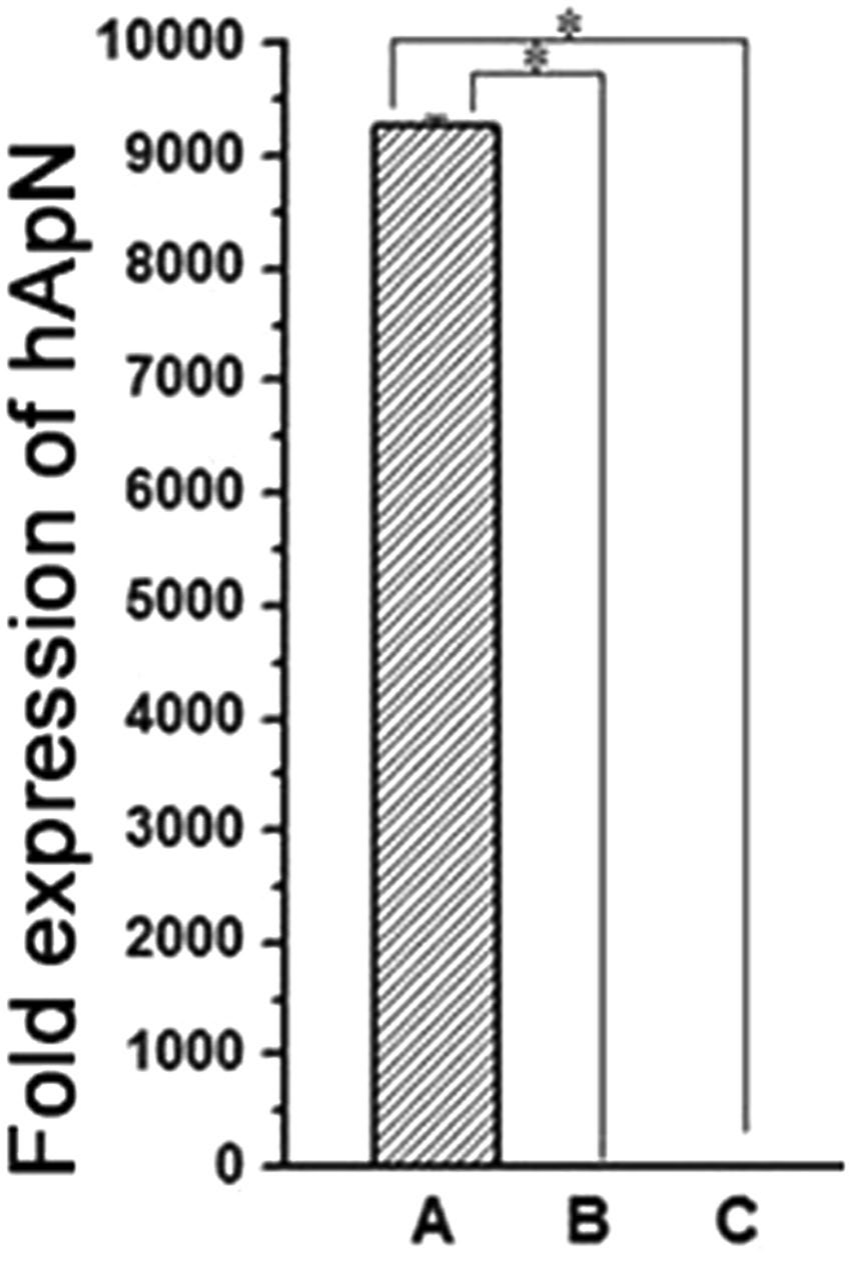
Gene expression of hAPN in BMSCs. High expression of hAPN in group A (Ad-hAPN-EGFP) and no expression in group B (Ad-EGFP) and group C (blank control). *P < 0.05.

Thirty-six hours after transfection, several EGFP + cells were counterstained with DAPI and observed and counted under fluorescence microscopy (Fig. 2). The transfection efficiency was as high as 95%. The PCR results of both our *in vivo* and our *in vitro* experiments showed high hApN expression in group A (Ad-hApN-EGFP) and no hApN expression in group B (Ad-EGFP) and group C (blank control) (Fig. 3 ). All of these findings indicated that the recombinant adenovirus was successfully transfected into the BMSCs and that the target genes were expressed at their normal levels *in vitro* and *in vivo*.

### AdipoR1 and AdipoR2 expression in BMSCs

**Figure 4:**
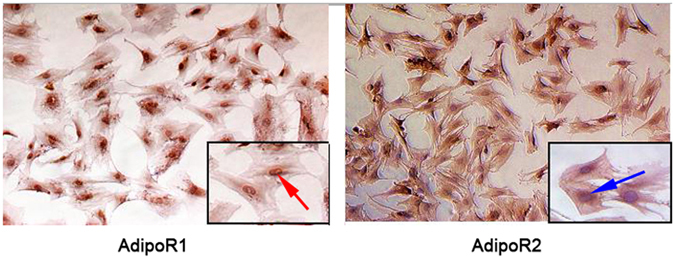
AdipoR1 and AdipoR2 expressed in BMSCs. AdipoR1 and AdipoR2 were dyed brown (red arrow), and the nucleus was stained with haematoxylin (blue arrow). AdipoR1 was expressed in the cytoplasm, cytomembrane and nucleus, while AdipoR2 was expressed only in the cytoplasm and cytomembrane.

The immunohistochemistry results are shown in Fig. 4. Uniformly distributed brown markers for AdipoR1 were observed throughout the cell, indicating that AdipoR1 was expressed not only in the cytoplasm and cytomembrane but also in the nucleus. In contrast, brown markers for AdipoR2 were observed merely in the cytoplasm and cytomembrane. AdipoR1 and AdipoR2 expressions were observed in BMSCs, indicating that ApN may act directly on BMSCs.

### hApN enhances osteogenesis through the Wnt/β-catenin pathway *in vitro*

**Figure 5:**
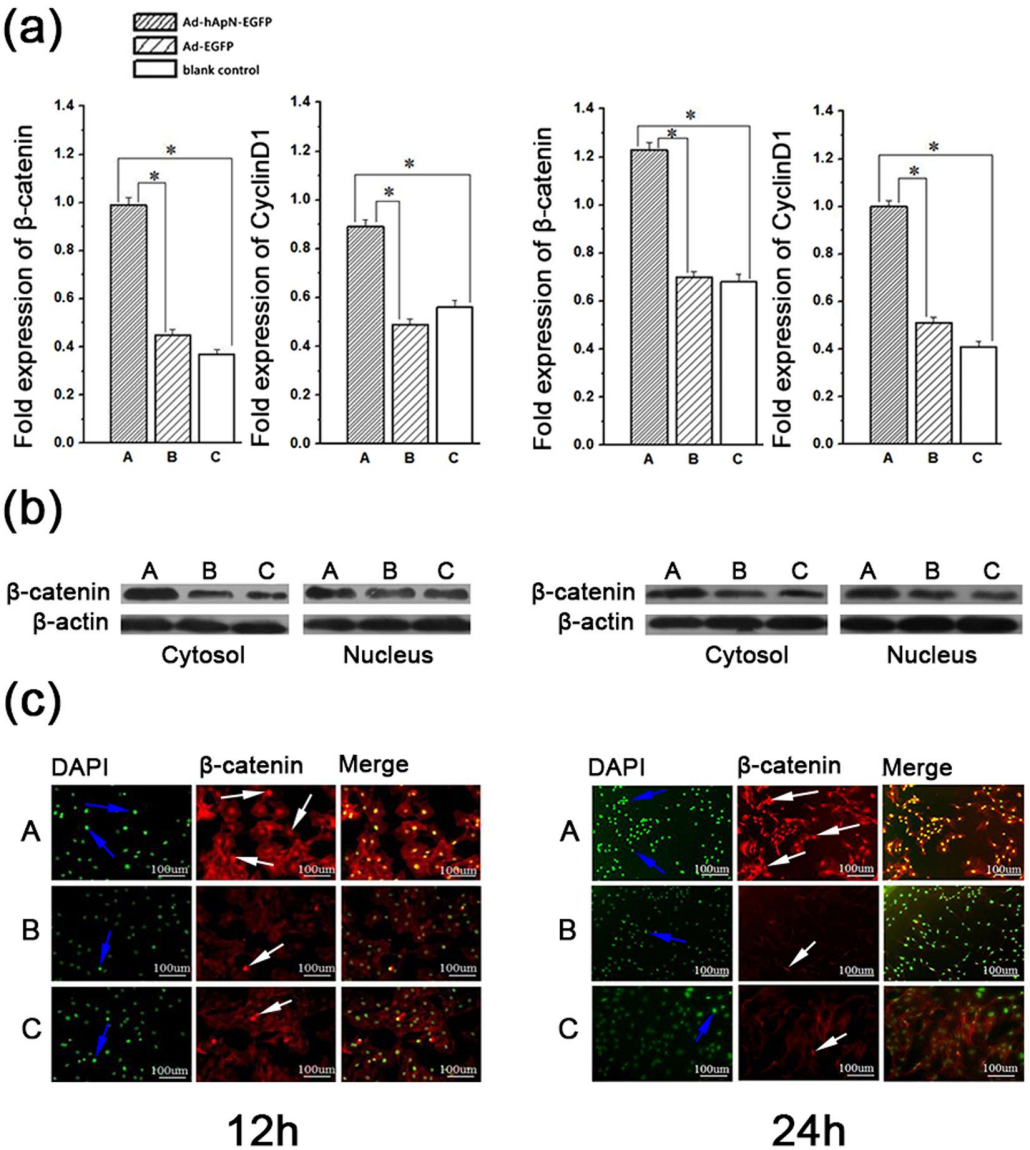
Gene and protein expressions of Wnt/β-catenin pathway related factors in BMSCs after osteogenic induction for 12 h (left side of Figure 5) and 24 h (right side). (**a**) Gene expressions of β-catenin and cyclinD1 by real-time PCR. Both genes were signifcantly elevated in group A (Ad-hApN-EGFP). *P < 0.05. (**b**) Protein expressions of β-catenin and cyclinD1 by western blotting. The two genes were up-regulated in group A both in cytoplasm and nucleus, and more β-catenin translocated into nucleus. (**c**) Immunofluorescence staining of BMSCs. Nucleus was stained with DAPI (blue arrow). The β-catenin (white arrow) expressed in both cytoplasm and nucleus and the fluorescent density and intensity were higher in group A.

To study the effects of hApN on Wnt/β-catenin pathway, we detected the mRNA and protein expression levels of the Wnt/β-catenin pathway-related factors β-catenin and cyclinD1 using real-time PCR, western blotting and immunofluorescence staining. After 12 h of osteogenic induction, the gene expression levels of both β-catenin and cyclinD1 were significantly elevated in group A (Ad-hApN-EGFP) compared with group B (Ad-EGFP) and group C (blank control) (Fig. 5a). The elevations in the expression levels of these two genes persisted until 24 h after osteogenic induction, at which time they were found to be greater than those noted at 12 h after osteogenic induction. The western blotting results for β-catenin and β-actin are displayed in Fig. 5b. β-actin served as an internal control to adjust the loading quantities of the protein sample. As expected, the protein expression levels of β-catenin in both the cytoplasm and the nucleus were much higher in group A than in groups B and C, as demonstrated by an analysis performed using ImageJ, indicating that β-catenin was translocated into the nucleus in the presence of hApN. Figure 5c shows the results for β-catenin immunofluorescence staining. The nucleus was stained with DAPI. Whether 12 h or 24 h after osteogenic induction, fluorescence density and intensity were obviously higher and stronger in group A than in groups B and C. Furthermore, β-catenin protein was expressed not only in the cytoplasm but also in the nucleus, a result consistent with the those of the western blotting analysis.

**Figure 6:**
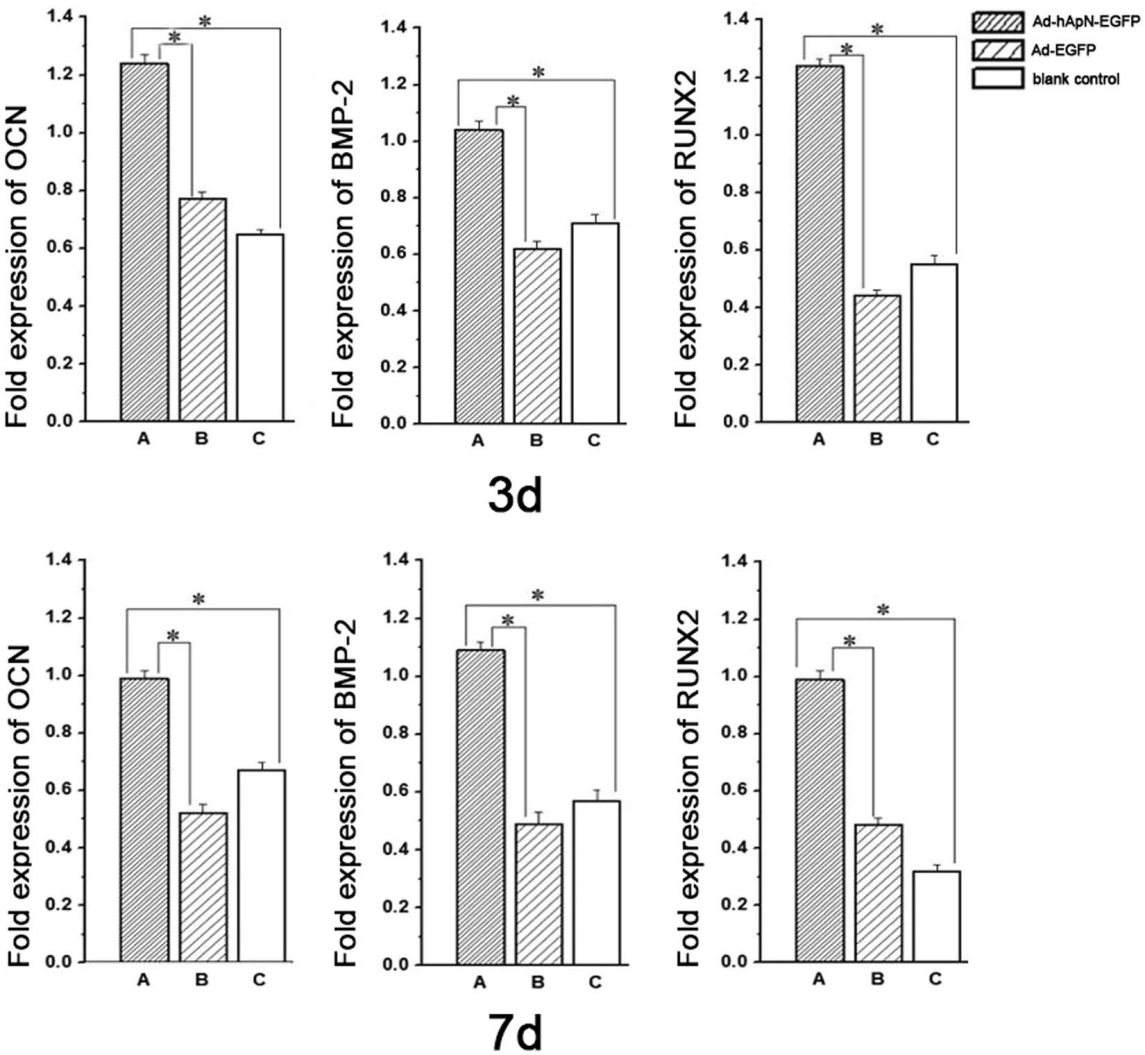
Osteogenesis detection of BMSCs. The expressions of osteogenic genes, including OCN, BMP-2 and RUNX2, were highly increased in group A (Ad-hApN-EGFP) compared with group B (Ad-EGFP) and group C (blank control) after osteogenic induction for 7 days. *P < 0.05.

**Figure 7:**
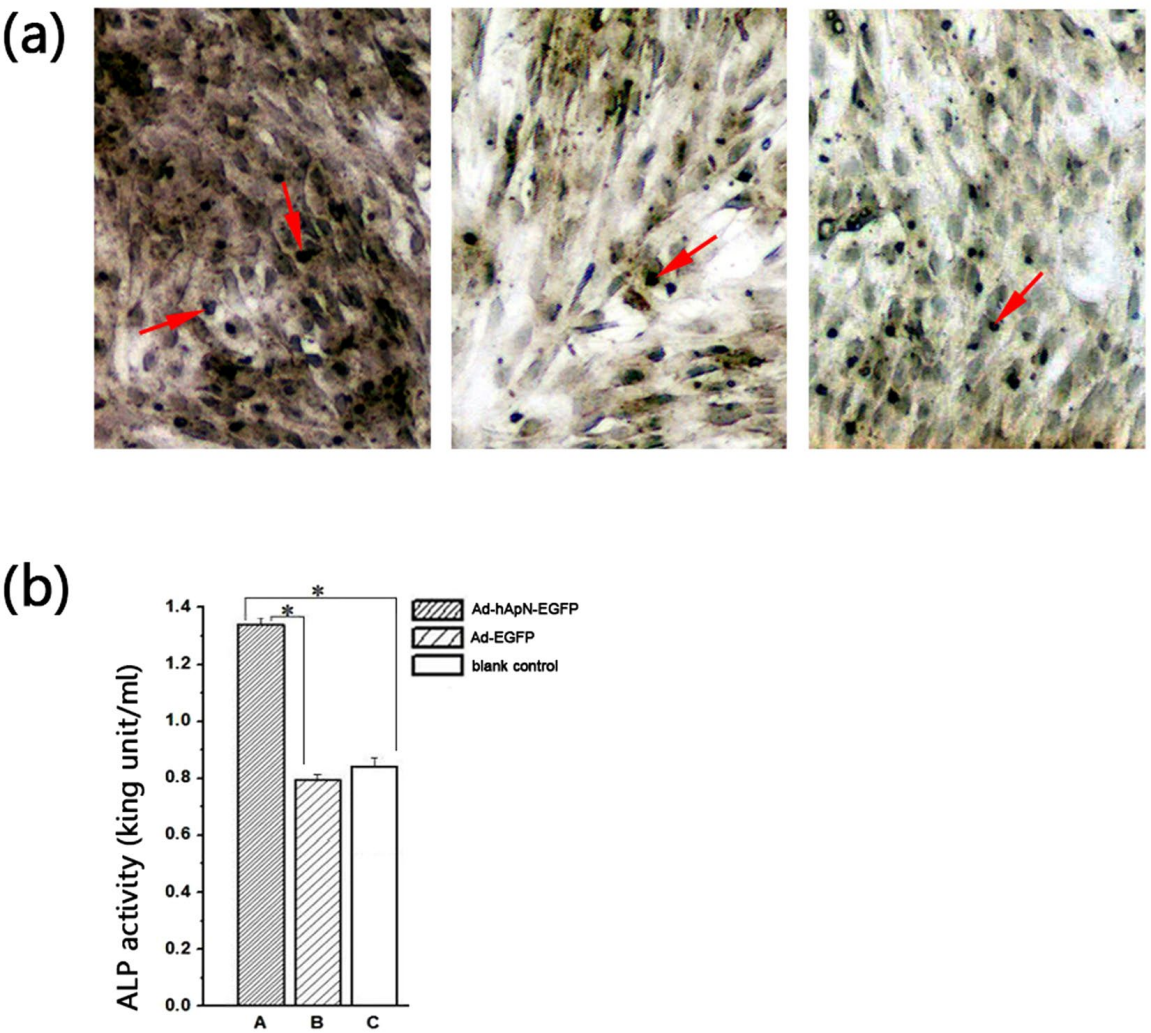
ALP staining/activity assays. (**a**) ALP staining was visualized under microscopy. More black cobalt sulfide precipitation (red arrow) were formed in Group A; (**b**) Significantly higher ALP activity was shown in group A compared with the other two groups (p < 0.05).

**Figure 8:**
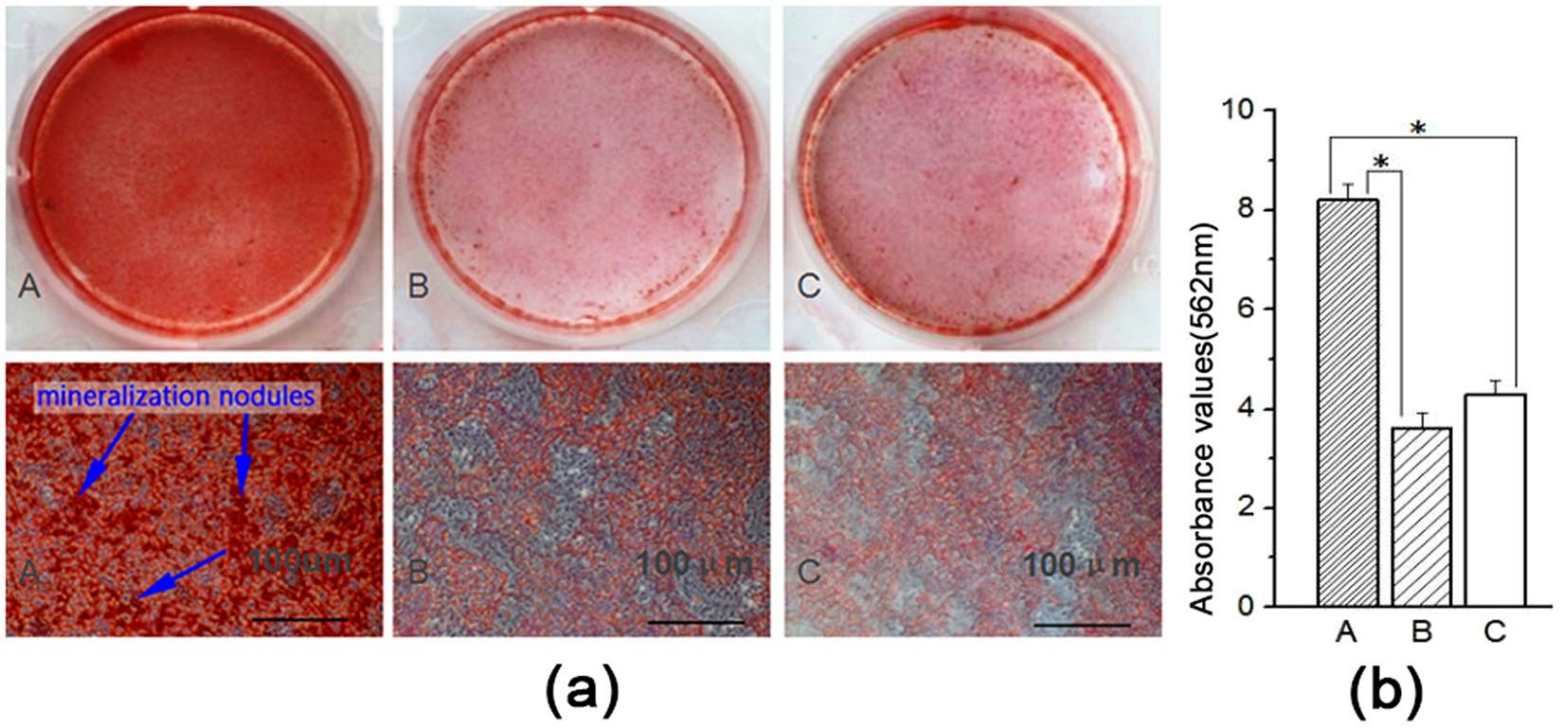
Alizarin Red staining and mineralization assays. (**a**) Extracellular matrix mineralization nodules (blue arrow) of group A were stained intensively and increased visibly; (**b**) Quantitative mineralization deposition of BMSCs.

After 3 d and 7 d of osteogenic induction, we detected osteogenic gene expression levels by real-time PCR. The levels of these three genes, namely, osteocalcin (OCN), bone morphogenetic protein-2 (BMP-2) and Runt-related transcription factor-2 (RUNX2), were markedly increased in group A compared with the other two groups (Fig. 6). After osteogenic induction for 14d, alkaline phosphatase (ALP) staining was visualized under microscopy (Fig. 7a). More black cobalt sulfide precipitation were formed in group A compared with the other two groups. The ALP activity was shown in Fig. 7b. Cells cultured with Ad-hApN-EGFP displayed significantly higher ALP activity compared with groups without hApN (p < 0.05). Calcium deposition, which served as a marker of the late stage of osteogenesis, was measured by alizarin red staining after 20 d of osteogenic induction. Significantly increased numbers of extracellular matrix mineralization nodules were observed in group A compared with groups B and C, and the staining intensity of group A was much stronger than those of groups B and C (Fig. 8a). The relative value of matrix mineralization in group A was enhanced by approximately 285% and 186% compared with those in groups B and C, respectively (Fig. 8b). Taken together, these results suggest that hApN enhanced osteogenesis through the canonical Wnt pathway *in vitro*.

### hApN enhances osteogenesis through the Wnt/β-catenin pathway *in vivo*

**Figure 9:**
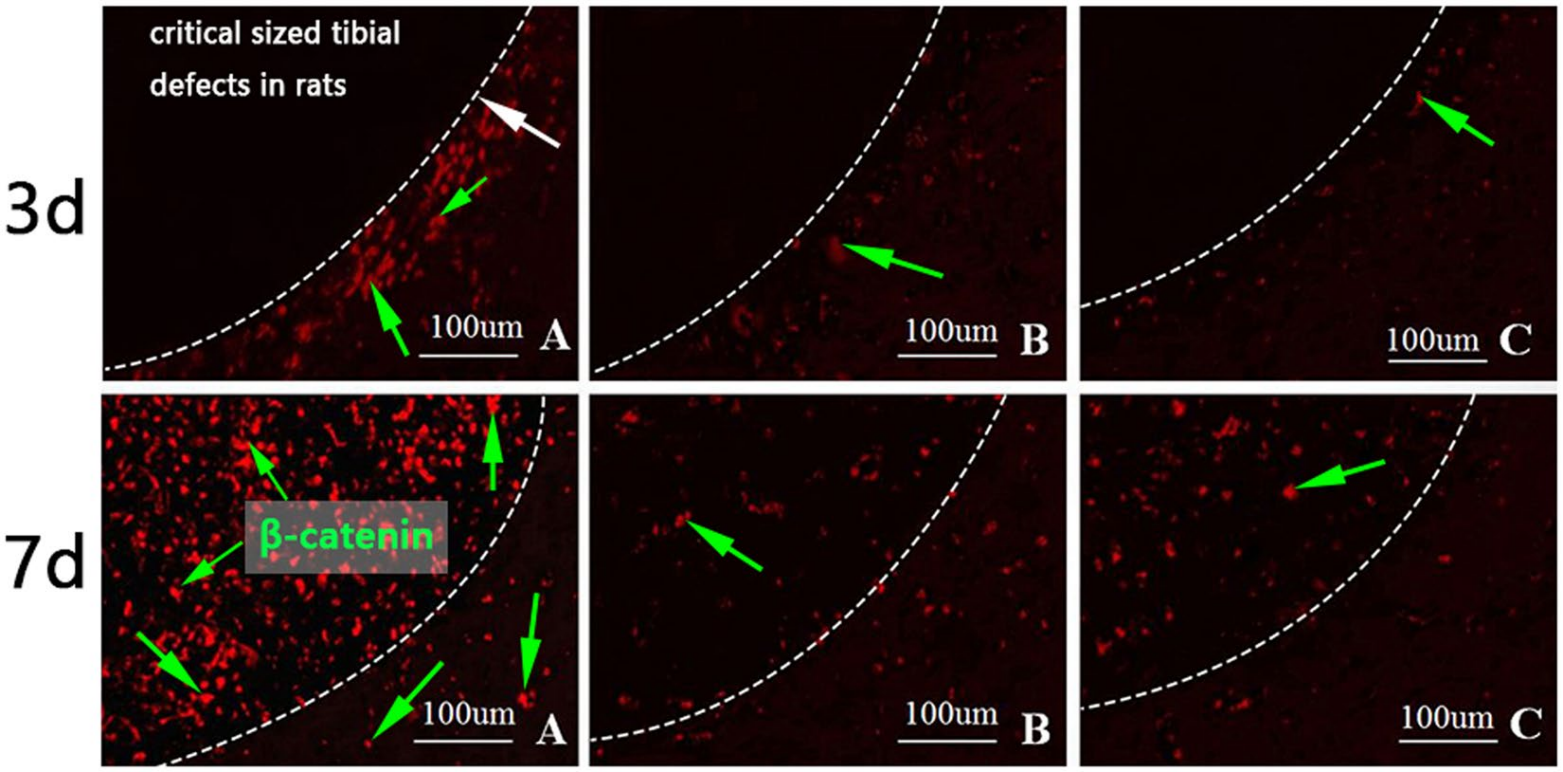
Immunofluorescence staining for β-catenin (green arrow). The white arrow indicated the bone defect edge. In group A, punctate red fluorescence was detected near the bone defect edges on day 3 and it was observed in bone defect areas on day 7. The fluorescence intensity and density of group A were obviously stronger than the other two groups.

In the animal experiment, we used real-time PCR and immunofluorescence staining to detect Wnt/β-catenin pathway-related gene expression on the 3rd and 7th postoperative days. β-catenin and cyclinD1 mRNA expression levels were evidently increased in group A compared with the other two groups at the above time points. Moreover, β-catenin expression levels were elevated in all three groups on the 7th postoperative day. The immunofluorescence staining results for β-catenin are shown in Fig. 9. On the 3rd day after surgery, we observed obvious punctate red fluorescence around the edge of the bone defect in group A, while the fluorescence signals in groups B and C were very weak. On the 7th postoperative day, we noted that the red fluorescence signal was widely dispersed throughout the entire bone defect area in group A, and the fluorescence intensity and density were obviously stronger in group A than in the other two groups.

**Figure 10:**
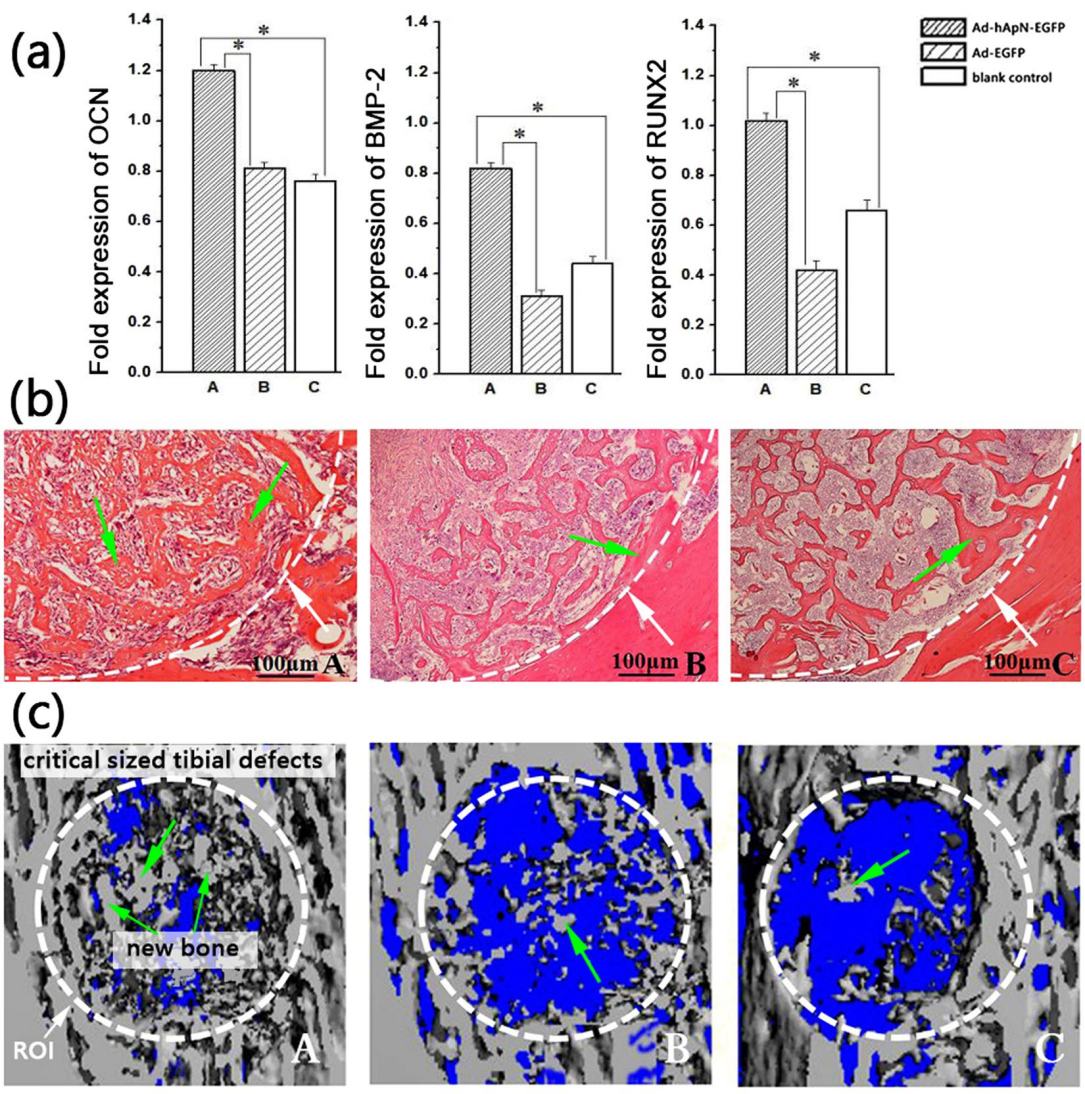
Osteogenesis detection in bone defect areas. (**a**) The expression of osteogenesis related genes on the 7th postoperative day, including OCN, BMP-2 and RUNX2. These three genes were significantly increased in group A compared with the other two groups. *P < 0.05; (**b**) Haematoxylin and eosin staining of bone defect areas on the 7th postoperative day. The white arrow indicated the bone defect edge. More new trabecular bone (green arrow) in group A; (**c**) Three-dimensional micro-CT images of bone defect areas. The white line dotted the region of interest area. More new bone (green arrow) were found in group A.

We detected the osteogenic effects of hApN by real-time PCR, HE staining and micro-CT. On the 7th day after surgery, the expression levels of the osteogenic genes, namely, OCN, BMP-2 and RUNX2, were significantly elevated in group A compared with groups B and C (Fig. 10a). The HE staining images are shown in Fig. 10b. The bone defect areas were filled with new bone in all three groups, but the amount and density of newly formed bone were significantly increased in group A compared with the other two groups. Quantitative analysis of HE staining showed that the ratios of new bone area to total tissue area in the three groups were 55.4%, 32.5% and 25.6%, respectively, and that the differences in the ratios between group A and the other two groups were statistically significant (*P* < 0.05). Three weeks after surgery, micro-CT was used to scan the new bone within the defects, and three-dimensional reconstruction was performed. Incomplete bone healing was observed, and blank areas without newly formed bone were noted in all three groups; however, the arrangement of trabecular bone was more dense, and fewer blank areas were noted in group A than in groups B and C (Fig. 10c). BMD, bone value/tissue value (BV/TV), trabecular number (Tb.N) and trabecular thickness (Tb.Th) were quantitatively analysed by selecting the regions of interest. As expected, the above parameters were highest in group A (Table 1).

**Table 1:** Microarchitecture parameters measured by micro-CT.

Bone indexes	Group A	Group B	Group C
BMD (mg.cm^−3^)	437	249	246
BV/TV (%)	42.23 ± 3.54	29.96 ± 2.78	26.48 ± 2.81
Tb.N (mm^−1^)	3.89	2.06	1.73
Tb.Th (μm)	98.2	51.4	54.6

## Discussion

To test the hypothesis that ApN could promote BMSC osteogenic differentiation and bone formation via the Wnt/β-catenin pathway, we detected AdipoR1 and AdipoR2 expression in BMSCs, constructed a recombinant adenovirus containing the hApN gene, and then transfected this adenovirus into BMSCs *in vitro* or injected it into bone defects *in vivo*. Finally, we detected Wnt/β-catenin pathway activity, osteogenesis related gene/protein expression and new bone formation through real-time PCR, western blotting, immunofluorescence, HE staining and micro-CT.

The immunohistochemistry results indicated that AdipoR1 and AdipoR2 were both expressed in the cytoplasm and cytomembrane of BMSCs and that AdipoR1 was also expressed in nucleus. These findings served as evidence that ApN can act on BMSCs directly. Real-time PCR showed higher levels of β-catenin, cyclinD1 expression in both the BMSCs and the bone defect areas that were treated with Ad-hApN-EGF. Importantly, more β-catenin translocated into nucleus and stabilized. β-catenin up-regulation in the nucleus activated the expression of downstream genes, such as cyclinD1, to exert multiple effects in different cells and tissues. The above results suggest that ApN is a potent facilitator of Wnt/β-catenin pathway activity. We also observed up-regulated expression of BMP-2, RUNX2 and OCN, which were the major factors in the differentiation of BMSCs into osteoblasts and the formation of bone matrix. The ALP staining showed more cobalt sulfide precipitation in group A, which means more alkaline phosphatase. Consistent with the staining, ALP activity assay showed higher ALP activity in group A. Alkaline phosphatase is a marker of early differentiation of osteoblasts, while OCN which mainly expressed during mineralization marks the osteoblast maturation. The expression of ALP and OCN implies the process of bone formation. Additionally, more extracellular matrix mineralization nodules were observed *in vitro*, and more newly formed bone was noted in bone defect areas *in vivo*. Quantitative analysis of BM, BV/TV, Tb.N and Tb.Th indicated that greater bone mass was present in bone defect areas in the presence of Ad-hApN-EGF. Thus, we surmised that ApN could enhance bone formation, whether *in vivo* or *in vitro*. In conclusion, we verified that ApN could promote BMSC osteogenic differentiation and bone regeneration, moreover, the Wnt/β-catenin pathway was involved in osteogenic effect of ApN.

We constructed a recombinant adenovirus containing hApN gene to create adiponectin autocrine and paracrine pathways. Based on our results, we deduced that ApN can enhance osteogenesis by exerting autocrine/paracrine effects, a hypothesis supported by the results obtained by Shinoda, who investigated the effects of adiponectin deficiency or overexpression on bone metabolism by establishing cell and animal models. The authors of that study concluded that (1) ApN exerts equivalent and balanced endocrine and autocrine/paracrine effects on bone, (2) ApN stimulates effective osteogenesis through its autocrine and paracrine effects, and (3) circulating ApN exerts direct/negative and indirect/positive effects on bone formation^12^.

ApN is abundant in plasma^35^, and a negative correlation between serum adiponectin level and bone mineralization has been reported in some studies^36^. Jurimae *et al*. noted lower bone mineral density and content in women with high circulating ApN level^37^. Luo *et al*. discovered that circulating ApN indirectly promoted osteoclast formation by suppressing osteoprotegerin production and stimulating RANKL^38^. These findings are not completely in conflict with our conclusions—which were based on Shinoda’s research—that circulating ApN and local ApN exert opposing effects on osteogenesis. However, the mechanism underlying this phenomenon is not clear yet.

ApN and AdipoR1 were discovered to be ubiquitously expressed in bone-forming cells^10^, indicating that ApN not only influences bone metabolism through its endocrine effects but also acts directly on bone-forming cells through its autocrine/paracrine effects^19, 23–26^. Chen *et al*. found ApN can activate the APPL1-AMPK pathway to promote osteogenic differentiation in human adipose-derived stem cells^23^. Lee *et al*. discovered that ApN promoted osteogenic differentiation in a cyclooxygenase-2-dependent manner in BMSCs and C3H10T1/2 cells^24^. Luo *et al*. showed that ApN enhanced human osteoblast differentiation and proliferation through the MAPK signalling pathway^26^. Pu *et al*. found that ApN can promote human jaw bone marrow mesenchymal stem cell osteogenesis through the APPL1-mediated p38 MAPK pathway and increase ossification-related gene expression levels^27^. However, very few studies have investigated the association between ApN and the Wnt/β-catenin pathway, one of the most important pathways in osteogenesis, with respect to the regulation of bone formation and BMSC osteogenic differentiation.

BMSCs are multipotent cells that facilitate regeneration of bone and other mesenchymal tissues^39^. Once a bone fracture occurs or a bone defect develops, special factors, such as SDF-1, are released by injured tissues to mobilize BMSCs throughout the entire body. Then, BMSCs recruit and migrate to the injury sites^40^ while proliferating and differentiating into osteoblasts^41–43^. Yu *et al*. established a calvarial bone defect model in mice and found that an increased number of BMSCs egressed into the peripheral blood in ApN-infused mice compared with wild-type mice and that more new bone formation was observed in the former group of mice than in the latter group of mice^44^. They showed that ApN can facilitate BMSC recruitment and migration, but they did not explore whether it can promote BMSC osteogenic differentiation. Lin *et al*. eliminated the BMSC AdipoR1 gene, which resulted in decreased β-catenin expression, decreased osteogenesis-related gene expression, and reduced mineralization. In contrast, higher bone mineral density was observed in AdipoR1 transgenic mice^25^. Based on the above evidence and the findings of the present study, we believe that ApN can facilitate new bone formation and BMSC osteogenic differentiation via the Wnt/β-catenin pathway.

More than 50 types of proteins are involved in the Wnt/β-catenin pathway. These proteins mediate the biological responses of various cells and play an important role in human embryonic development and in regulating the internal environment of the human body^45, 46^. Therefore, the Wnt/β-catenin pathway not only participates in bone metabolism but also takes part in some diseases. Gene abnormalities affecting the Wnt/β-catenin pathway can induce colon cancer^47^, hair follicle tumours^48^, leukaemia^49^, mammary cancer^50^ and some other diseases. Interestingly, ApN was found to attenuate mammary tumourigenesis by stimulating Wnt inhibitory factor-1 and suppressing the Wnt/β-catenin pathway in human breast cancer cells^51, 52^. The Wnt/β-catenin pathway has also been reported to be involved in tissue and organ fibrotic processes, such as myocardial fibrosis^53^, pulmonary fibrosis^54, 55^ and hepatic fibrosis^56^, while ApN has been shown to have anti-fibrosis activity^57^. Reinke *et al*. showed that ApN can inhibit the phosphorylation of Lrp6, a co-receptor for Wnt protein^58^, thus suppressing the Wnt/β-catenin pathway in human dermal fibroblast^59^. Therefore, we speculate that a connection exists between the protective effects of ApN against tissue fibrosis and the negative actions of ApN on the Wnt/β-catenin pathway. However, in another study, the expression levels of β-catenin and the downstream genes of the canonical Wnt pathway were neither up-regulated nor down-regulated in the adipose tissue of ApN-transgenic mice compared with wild-type mice^60^. The above studies showed that ApN had three different effects on the canonical Wnt pathway, an inhibitory effect in human breast cancer cells and dermal fibroblasts, no influence in adipose tissue and promontory effect in BMSCs.

It is noteworthy that Reinke *et al*. found that ApN can inhibit β-catenin in AdipoR1/R2 gene knock-out fibroblasts^59^, indicating that ApN can regulate the canonical Wnt pathway through some other unknown receptors in addition to AdipoR1/R2. However, the internal environment of the body is complex and adaptable, and the factors affecting the canonical Wnt/β-catenin pathway and bone formation are more intricate. Therefore, there is still a need to explore the effects of ApN on the Wnt/β-catenin pathway further.

Bone defect is a challenging problem for clinicians. Identifying and developing efficient and safe methods for treating bone defects remain areas of focus for researchers. Gene therapy is a new technology used to treat diseases at the DNA level that has been shown to have long-term efficacy^61^. A variety of studies using gene therapy for bone defects have shown satisfactory results; however, the experiments were limited to animals. More safety and technical issues related to this therapy remain to be resolved, and an effective, secure and economical approach of administering it is still being sought. Nevertheless, gene therapy still has great clinical potential for the treatment of bone diseases.

In summary, the findings of the present study indicate that ApN could act on BMSCs and confirm the hypothesis that ApN could promote BMSC osteogenic differentiation and bone formation through the Wnt/β-catenin pathway in both ApN gene-modified BMSCs and ApN gene-transgenic mice. Our findings indicate that ApN may represent a strategy for treating bone defects and metabolic bone diseases. And in subsequent research, gene knock-out experiment is needed to further confirm the importance of Wnt/β-catenin pathway in the osteogenic effect of ApN.

## Methods

### Adenovirus construction

Recombinant plasmids containing the human adiponectin (hApN) fragment were obtained from Tufts University School of Dental Medicine. Recombinant adenoviruses containing the hApN and enhanced green fluorescent protein (EGFP) genes were constructed and identified as described in previously published studies^62^. Ad-hApN-EGFP plaque forming units (PFUs) were 1 × 10^10^ per millilitre.

### Cell culture

Sprague-Dawley (SD) rat primary BMSCs were isolated using the methods described by Friedenstein^41^. Then, the cells were cultured in α-MEM supplemented with 10% foetal bovine serum (Sigma-Aldrich, St. Louis, MO,USA) at 37 °C. The medium was replaced every three days. After the cells reached 90% confluence, they were digested with trypsin enzymes for passaging. The third BMSC passage was incubated with a fluorescently labelled antibody (Beyotime, Shanghai, China) for 30 min and then washed with PBS. The expression levels of different cell surface markers, including CD29, CD90, CD34, and CD45, were detected by flow cytometry. Cells from the third passage were used in subsequent experiments.

### Immunohistochemistry for AdipoR1 and AdipoR2 expression in BMSCs

Third-generation BMSCs were seeded into a 24-well plate. Cell adherence was observed after 12 h. Following several washes with PBS, the cells were fixed in 4% paraformaldehyde at room temperature, washed with PBS again, and permeabilized with 0.5% Triton X-100 for 20 min. A Plink-2 plus Polymer HRP Detection System (GBI Labs, Mukilteo,WA, USA) and DAB Horseradish Peroxidase Color Development Kit (Beyotime) were used to detect AdipoR1 and AdipoR2 expression in BMSCs, according to the manufacturer’s instructions. The nuclei were then stained with haematoxylin again. The concentrations of the primary antibodies for AdipoR1 and AdipoR2 were 1:200 and 1:400, respectively.

### Ad-hApN-EGFP transfection and BMSC osteogenic induction

The third BMSC passage was cultured in a 6-well plate at a density of 1 × 10^5^ cells per millilitre. Half of the medium was changed after 24 hours. The cells were separated into the following three groups: group A (Ad-hApN-EGFP), which was incubated with 5 µl of recombinant adenovirus solution (1 × 10^10^ PFU/ml); group B (Ad-EGFP), which was incubated with 5 µl of adenovirus solution containing no adiponectin; and group C (blank control), which was incubated with an equal volume of serum-free medium. The multiplicity of infection (MOI) was 50. After 36 h of transfection, we used fluorescence microscopy to scan the cells, which were stained with DAPI, and calculated the transfection efficiency of the recombinant adenovirus. Then, the medium was replaced with an osteogenic induction solution and renewed every three days. Samples were collected and analysed at 12 h, 24 h, 3 d, 7 d and 20 d after osteogenic induction.

### Animal model of bone defects

All procedures were ratified by the Animal Care Committee of Sichuan University. All the experiments were carried out in accordance with the relevant guidelines and regulations.A total of 72 Sprague-Dawley rats were anaesthetized. Then, a longitudinal incision was made through the skin, subcutaneous tissue and periosteum along the medial aspect of the knee joint to expose the tibia. Along the medial aspect of each tibia, we used a dental engine (APS, Wallingford,CT,USA) to create a critical 2-mm-in-diameter bone defect located one millimetre away from the growth plate. The bone marrow cavity was exposed, but the contralateral cortical bone structures remained intact. The defect was closed in layers after the procedures. All rats were intramuscularly injected with 400 thousand units of penicillin postoperatively and twice a day for three consecutive days. The rats were randomly allocated into following three equal groups: group A (Ad-hApN-EGFP), which received 5 µl of adenovirus solution (pfu 1 × 10^10^/ml) that was diluted with 0.25 ml of normal saline and injected into the bone defect of each tibia during surgery and on the 2^nd^ post-operative day; group B (Ad-EGFP), which was treated with 0.25 ml of virus solution containing no adiponectin. The solution was injected into the bone defect at the above time points; group C (blank control), which was treated with the same dose normal saline. The dose was injected by the same method and at same time point as those indicated above. On days 3, 7, and 21 after surgery, the rats were killed with a narcotic overdose. The tibias were quickly removed under aseptic conditions and then preserved in liquid nitrogen after soft tissue removal and several washes with normal saline.

### BMSC osteogenic differentiation

#### Real-time PCR (RT-PCR)

Osteogenic gene expression levels in BMSCs were assessed as follows: total cellular RNA was extracted using Trizol Reagent (Invitrogen, CA, USA) after 12 h, 24 h, 3 d and 7 d of osteogenic induction. Then, cDNA was synthesized using a PrimeScript RT Reagent Kit with a gDNA Eraser (Takara, Tokyo, Japan), according to the manufacturer’s instructions. Gene expression levels were quantified by an ABI PRISM 7300 (Applied Biosystems, Foster City, USA) with SYBR® Premix Ex Taq™ II (Takara). The following PCR cycling protocol was used: 95 °C for 20 s, followed by 40 cycles of 95 °C 10 s and 60 °C 30 s. The expression levels of the Wnt/β-catenin pathway-related genes β-catenin and cyclinD1 were detected after 12 h and 24 h of osteogenic induction. On the 3rd and 7th day after osteogenic induction, we detected the expression levels of RUNX-2, BMP-2 and OCN. Table 2 lists the sequences of the specific primers used in the study. GAPDH was used as a control, and all primers were purchased from Life Technologies Corporation, ThermoFisher (USA).

**Table 2:** Primers used for RT-PCR.

Gene	Forward primer	Reverse primer
hApN	GCTCTGTGCTCCTGCATCTG	GAGTCCATTACGCTCTCCTTCC
OCN	GGTGGTGAATAGACTCCGGC	GCAACACATGCCCTAAACGG
BMP-2	CGCCTCACAAACAACCACAG	AATGACTCGGTTGGTCTCGG
RUNX-2	AGAATGGACGTGCCCCCTA	CTGGGGAAGCAGCAACACTA
β-catenin	TGGCAACCAAGAAAGCAAG	CTGAACAAGAGTCCCAAGGAG
cyclinD1	CCCTCGGTGTCCTACTTCA	GTTTGTTCTCCTCCGCCTCT
GAPDH	TCCATGACAACTTTGGTATCG	TGTAGCCAAATTCGTTGTCA

#### Western blotting

After 12 hours and 24 hours of osteogenic induction, the adherent cells were washed with PBS, isolated by scraping, and centrifuged at 10000 rpm for 8 min, Then, BMSC cytoplasmic and nuclear proteins were isolated using a Nuclear and Cytoplasmic Protein Extraction Kit (Beyotime). Protein concentrations were detected using a BCA Protein Assay Kit (Sigma-Aldrich). The proteins were subjected to SDS-PAGE on an 8% polyacrylamide gel and then transferred onto a PVDF membrane (Sigma-Aldrich) and incubated with 5% nonfat milk for 1 h before being washed thrice with TBST (10 mM Tris-HCL (pH 7.6)). The membranes were then incubated at 4 °C overnight with a rabbit anti-rat primary antibody for β-catenin before being incubated with an HRP-labelled secondary antibody. After 1 h, the protein bands were visualised via chemiluminescence using an ECL kit (Thermo Scientific, Waltham, MA, USA). Protein quantification was performed using ImageJ.

#### Immunofluorescence

The BMSCs were seeded in a 24-well plate for 12 h of osteogenic induction, fixed in 4% paraformaldehyde for 10 min, permeabilized with 0.25% Triton X-100 in 10% normal goat serum for 30 min, and then incubated with the indicated primary antibody (rabbit anti-rat β-catenin, 1:150, Beyotime) overnight at 4 °C. The following day, after being washed thrice with PBS, the cells were cultured with the appropriate secondary antibody (1:100), which was conjugated with TRIT for 30 min at room temperature. After being stained with DAPI, the cells were observed via fluorescence microscopy (Olympus, Japan).

#### ALP staining/activity assays

After osteogenic induction for 14 d, cells were fixed with 4% paraformaldehyde for 2 min, then washed with PBS, stained by ALP staining kit mixture (Leagene Biotech, Beijing, China) according to the instructions,then visualized under microscopy. To quantify ALP activity, cells were disrupted by RIPA Lysis Buffer (Beyotime). After centrifugation, the supernatant was used to detect ALP activity by Alkalin Phosphatase Colorimetric Assay Kit (Leagene Biotech).The optical density was measured at 405 nm.

#### Alizarin Red staining

The BMSCs were cultured in 24-well plates to undergo osteogenic induction for 20 d before being fixed in 95% ethanol for 30 min and stained with 1% (w/v) AR-S (pH 4.2) for 5 min. After the cells had been washed 3 times with ddH_2_O, we visualized calcium deposits by inverted phase-contrast microscopy (Olympus, Japan). The Alizarin Red-stained cells were cultured with 10% cetylpyridinium chloride solution for 25 min to quantify matrix mineralization. The absorbance of the supernatant was then measured at 562 nm.

### SD rat bone formation

#### Real-time PCR

On the 3rd and 7th postoperative day, tibia samples were harvested, cracked and ground into powder. Total RNA was extracted using Trizol reagent, and then cDNA was synthesized using a PrimeScript RT Reagent Kit with gDNA Eraser, according to the manufacturer’s instructions. OCN, BMP-2 and RUNX-2 gene expression levels were quantified by an ABI PRISM 7300 with SYBR® Premix Ex Taq™ II. The primers used for the experiment are listed in Table 1.

#### Immunofluorescence

On the 3rd and 7th postoperative day, tibia samples were fixed in 4% paraformaldehyde for 48 h. After several washes with PBS, the samples were decalcified using 10% EDTA decalcifying fluid (Sigma-Aldrich) for 60 d, dehydrated in graded alcohol, embedded in paraffin and cut into 5-μm-thick sections. After antigen retrieval and blocking with 10% normal goat serum for 20 min, the samples were cultured with rabbit anti-rat β-catenin (1:150) overnight at 4 °C. Then, the samples were incubated with the appropriate secondary antibody for 30 min~60 min at 37 °C before finally being evaluated with a fluorescence microscope.

#### HE staining

On the 7th postoperative day, we obtained 5-μm-thick paraffin sections and stained them with haematoxylin and eosin. We used light microscopy (100x) to scan the samples and collect images. The ratio of the new bone area to the entire sight area was calculated.

#### Micro-CT

Three weeks after surgery, tibia samples were harvested, fixed in 4% paraformaldehyde, and scanned by micro-CT (u-CT80, SCANCO, Switzerland) (working voltage 70 kV, working current 114 uA, integration time 700 ms). Three-dimensional reconstruction was performed. BMD, BV/TV, Tb.N and Tb.Th were quantitatively analysed.

### Statistical analysis

All the data were expressed as the mean ± standard deviation and analysed using SPSS 19.0. Comparisons between groups were evaluated by one-way ANOVA. *P* values less than 0.05 were considered statistically significant.
